# Human Dental Pulp Stem Cells Exhibit Different Biological Behaviours in Response to Commercial Bleaching Products

**DOI:** 10.3390/ma11071098

**Published:** 2018-06-27

**Authors:** Carmen Llena, Mar Collado-González, Christopher Joseph Tomás-Catalá, David García-Bernal, Ricardo Elías Oñate-Sánchez, Francisco Javier Rodríguez-Lozano, Leopoldo Forner

**Affiliations:** 1 Department of Stomatology, University de Valencia, 46010 Valencia, Spain; llena@uv.es (C.L.); forner@uv.es (L.F.); 2 School of Dentistry/Cellular Therapy and Hematopoietic Transplant Unit, Hematology Department, Virgen de la Arrixaca Clinical University Hospital, IMIB-Arrixaca, University of Murcia, 30120 Murcia, Spain; mdmcg1@um.es (M.C.-G.); ctc20203@um.es (C.J.T.-C.); redond@gmail.com (D.G.-B.); reosan@um.es (R.E.O.-S.); 3 School of Dentistry, Hospital Morales Meseguer 2pl. Av. Marqués de los Vélez s/n, University of Murcia, 30008 Murcia, Spain

**Keywords:** bleaching products, diffusion, cytotoxicity, dental pulp, stem cells

## Abstract

The purpose of this study was to evaluate the diffusion capacity and the biological effects of different bleaching products on human dental pulp stem cells (*h*DPSCs). The bleaching gel was applied for 90, 30 or 15 min to enamel/dentine discs that adapted in an artificial chamber. The diffusion of hydrogen peroxide (HP) was analysed by fluorometry and the diffusion products were applied to *h*DPSCs. Cell viability, cell migration and cell morphology assays were performed using the eluates of diffusion products. Finally, cell apoptosis and the expression of mesenchymal stem cell markers were analysed by flow cytometry. Statistical analysis was performed using analysis of variance and Kruskal–Wallis or Mann–Whitney tests (α < 0.05). Significant reductions of approximately 95% in cell viability were observed for the 3 × 15 min groups (*p* < 0.001), while 1 × 30 min of PerfectBleach and 1 × 90 min of PolaNight resulted in reductions of 50% and 60% in cell viability, respectively (*p* < 0.001). Similar results were obtained in the migration assay. Moreover, the 3 × 15 min group was associated with cell morphology alterations and reductions of >70% in cell live. Finally, *h*DPSCs maintained their mesenchymal phenotype in all conditions. Similar concentrations of carbamide peroxide (CP) and HP in different commercial products exhibited different biological effects on hDPSCs.

## 1. Introduction

The first study examining the chemical mechanism of hydrogen peroxide (HP) when coming into contact with enamel was published in 1970, which demonstrated that HP reached the dentin structure to produce a whitening effect [1]. The permeability of hard dental tissues and the low molecular weight of HP explain HP diffusion [2,3]. The bleaching process occurs when the low molecular weight of HP diffuses through enamel and dentin as it releases reactive oxygen species (ROS) that react with other free or weakly bound substances before regaining molecular stability. This oxidant phenomenon may explain the complex mechanism of dental bleaching [4]. The presence of oxidising agents and the penetration capacity of HP are closely correlated with each other [5].

HP reaches the pulp chamber via the dentinal tubules, decreases cell metabolism and viability [6,7] and induces vascular permeability changes [8], DNA modifications and pulpal necrosis [9,10,11,12].

Dental pulp is a loose connective tissue that occupies the pulp chamber of the tooth, which originates from the embryonic dental papilla (ectomesenchymal tissue) [13]. It has a high capacity for repair since it exhibits a significant regenerative response in reaction to injury or trauma, which causes the secretion of tertiary dentine and promotion of the differentiation of dental pulp stem cells (*h*DPSCs) into odontoblast-like cells. In addition, *h*DPSCs are able to differentiate toward osteogenic, myogenic and adipogenic lineages, melanocytes, Schwann cells and neurons [14].

Previous studies have analysed the biocompatibility of several bleaching agents, although there are fewer investigations studying specific commercial products and the effect of different times of applications [15,16,17,18,19]. Furthermore, the different compositions of commercial bleaching products are often unknown and commercial bleaching products have provided differing results for their biocompatibility in vitro and in vivo.

Cell cultures are suitable for evaluating the effects of different dental products and materials on pulp tissues [20] and the biological response of human pulp cells to these products and materials. The results of these in vitro studies are not directly comparable to those in humans, but these studies provide a good model with which to analyse different products and techniques and their potential risks [21].

The literature has questioned whether the use of bleaching products with a high concentration of HP is necessary or even safe. However, these products have been applied and reapplied multiple times in the same clinical session in order to increase the speed of changing the colour of the teeth. Although the whitening effect is known to be related to the diffusion of peroxide through the dental tissues, studies suggest that the bleaching effect is not related to the constant reapplication of the gel because good results have been obtained with the technique of a single clinical application [22]. Thus, this finding might support the adoption of a new dosage that is based on a single application of the bleaching product. Thus, given that high concentrations of peroxide are potentially harmful to the pulp cells [7,23], a focus on the posology that is guided by the adoption of milder protocols is both appealing and justifiable in an effort to find safe alternatives to bleaching.

In addition to the discrepancy in the clinical application protocols, the diffusion of and pulp damage caused by different commercial products with similar HP concentrations is still not clear.

The present study evaluated the diffusion capacity and cytotoxicity of different high-concentration commercial bleaching products and different application protocols in vitro. The null hypothesis was that different commercial bleaching products with equal or similar concentrations of HP would not promote different responses in dental pulp stem cells. This study was conducted in order to determine if other substances contained in the product are responsible for the cytotoxicity of the commercial bleaching product.

There are no studies that have determined the toxicity of the excipients contained in different commercial bleaching products with equal or similar HP concentrations.

## 2. Materials and Methods

### 2.1. Specimen Selection and Preparation

We selected freshly impacted mandibular third molars (*n* = 25) from patients aged 18 to 35 years to obtain a homogeneous sample. Selected teeth were not subjected to the oral environment, occlusal loads or other functional aggressions. The study was approved by the Ethics Committee of the University of València (registration number H1443515306255).

Organic remains were removed from the teeth and the integrity of the dental structure was confirmed. Selected teeth were immersed in a 0.1% thymol solution and preserved at 4 °C for 48 h. Teeth were removed from the thymol solution and immersed in deionized water at 4 °C until use.

Twenty-five samples that contained 2 mm of enamel and 2 mm of dentin were prepared as follows. Each tooth was cut mesio-distally with a diamond disk mounted on a hand piece with water-cooling. Roots were discarded. The dentin surface was reduced with 400- and 600-grit silicon carbide paper Sof-lex™ discs (3M Dental Products, St. Paul, MN, USA) until the dentin thickness was 2 mm. The sample dimensions were in the range of 0.5–0.7 cm × 0.4–0.6 cm. The dentin surface was treated with 0.5 M ethylene diamine tetra-acetic acid (EDTA) at a pH of 7.4 for 30 s, which was subsequently rinsed with deionized water to remove the smear layer and stored in deionized water at 4 °C until use.

**Scheme 1:**
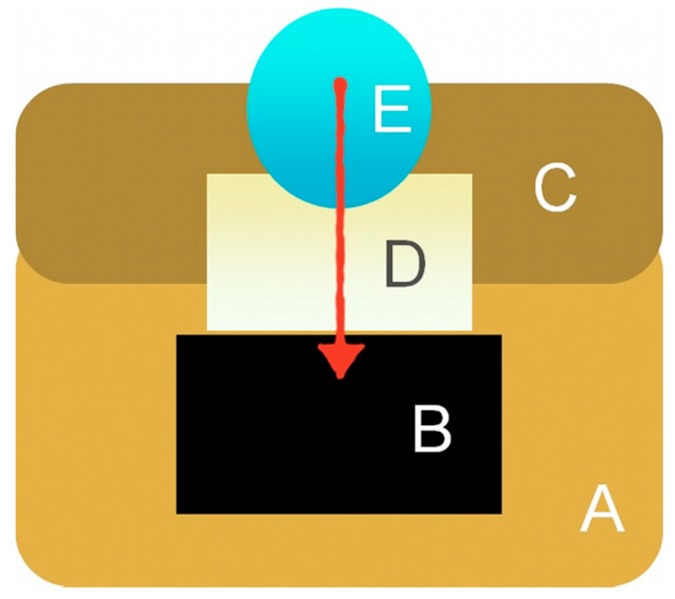
Schematic representation of the artificial pulp chamber. Heavy silicone (A,C) was utilized to stabilize the sample (D), maintain the buffer (B) and prepare the artificial pulp chamber.

An artificial pulp chamber was prepared with heavy silicone (Panasil R Putty, Kettenbach, Huntington Beach, CA, USA) with a capacity of 100 μL. To stabilize the sample, a heavy silicone ring was fabricated to anchor the sample, which allowed the dentin to be in contact with the buffer and had an upper window for placing the study gel (Scheme 1). This ring fit perfectly in the artificial pulp chamber, which maintained the fixation of the sample, while additional sealing was performed between the sample and the ring by using wax.

### 2.2. Diffusion Evaluation

The experimental specimens were randomized into five different groups (*n* = 5 each): group 1 was exposed to a neutral pH gel of 37.5% HP -Pola Office + (PO)- (SDI, Bayswater, VIC, Australia) for 30 min (PO30); group 2 was exposed to the same product as group 1 but with 3 applications of 15 min (PO3x15); group 3 was exposed to neutral pH gel of 35% HP -PerfectBleach (PB)- (Voco, Cuxhaven, Germany) for 30 min (PB30); group 4 was exposed to the same product as group 3 but with 3 applications of 15 min (PB3x15); and the group 5 was exposed to 16% carbamide peroxide (CP) -PolaNight (PN)- (SDI) for 90 min (PN90).

The diffusion of HP from different bleaching products was analysed using fluorimetry. The production of a homovanillic acid dimer from a reaction catalysed by peroxidase using HP as a substrate was measured as described by Barja [24]. Samples that had reacted with the peroxidase were measured using a fluorimeter (model F-4500 fluorescence spectrophotometer, Hitachi, Japan). A standard fluorimetry signal curve of HP (H1009-100ML, Sigma-Aldrich, St. Louis, MO, USA) was generated. The same dilutions were measured in a Helios Alpha UV−vis spectrophotometer (Thermo Fisher Scientific Inc., Waltman, MA, USA). Therefore, the fluorimetry signal was related to the concentration of the fluorescent dimer.

Phosphate-buffered saline (PBS; 400 μL) was placed in the buffer in the reservoir. Gel (0.5 μL) was applied to the external enamel surface and the film was coated (Parafilm M, Sigma-Aldrich, St. Louis, MO, USA). The HP in the PO15 and PB15 groups was replaced every 15 min without removal of the PBS from the reservoir. The HP remained for 30 min in the PO30 and PB30 groups and 90 min in the PN90 group. The PBS containing the diffused HP from the reservoir was removed. The bleaching agent application process was performed at 37 °C.

A volume of 100 μL of the sample containing the diffused HP was removed and added to 1400 μL of the reaction buffer. Glycine−EDTA buffer (500 μL) was added after 15 min. Fluorescence intensity was measured, before the concentration value was extrapolated from the standard curve from the controls.

### 2.3. Biological Assays

#### 2.3.1. Isolation and Culture of hDPSCs

hDPSCs were isolated and characterised as described previously [25]. Human DPSCs (hDPSCs) were obtained from impacted third molars collected from healthy subjects (*n* = 15). Donors provided written informed consent according to the guidelines of the Ethics Committee of our Institution. Briefly, the pulp chamber was exposed after removal of the tooth and the pulp tissue was retrieved, thoroughly minced and digested by means of a collagenase I (3 mg/mL) and dispase II (4 mg/mL) buffer (Invitrogen, Karlsruhe, Germany) for 45 min at 37 °C. The cells were cultured with a-MEM (Minimum Essential Media) medium (Invitrogen), supplemented with 15% fetal bovine serum (FBS, Invitrogen), 100 mM L-ascorbic acid phosphate (Sigma-Aldrich, Steinheim, Germany) and antibiotics/antimycotics (Complete Culture Medium, CCM) before incubating at 37 °C in 5% CO_2_. This study was performed using hDPSCs from 4th passage of the culture.

#### 2.3.2. Characterisation Assay

The mesenchymal immunophenotype of cultured human DPSCs was analyzed by flow cytometry. Single cell suspensions obtained by culture trypsinization were labelled or surface markers with fluorochrome-conjugated antibodies: CD73 (clone AD2), CD90 (clone DG3), CD105 (clone 43A4E1), CD14 (clone TÜK4), CD20 (clone LT20.B4), CD34 (clone AC136) and CD45 (clone 5B1) (Human Mesenchymal (MSC) Stem Cell Phenotyping Cocktail, Miltenyi Biotec, Bergisch-Gladbach, Germany), following the recommendations of the International Society of Cellular Therapy (ISCT) [26]. Flow cytometry analyses were performed using a FACSCanto II flow cytometer (BD Biosciences, San José, CA, USA).

#### 2.3.3. Conditioned Medium

Culture medium conditioned with the bleaching products (BPs) was obtained as described by Cavalcanti et al. [27] in compliance with the ISO 10993-12 [28] proceedings. Briefly, 100 μL of the sample containing the diffused HP was removed and was collected into aliquots, before dilutions of 1%, 0.5% and 0.25% were created using cell culture medium. The conditioned medium was filtered in a sterile environment immediately and was used for cell culture exposure.

#### 2.3.4. Cell Viability Assay

The metabolic activity of hDPSCs growing in the presence of different bleaching extracts was evaluated using the MTT assay (MTT Cell Growth Kit, Chemicon, Rosemont, IL, USA). hDPSCs were initially loaded with 1 × 10^3^ cells per well and a volume of 180 μL in 96-well plates. Cells were starved for 24 h in serum-free medium at 37 °C in a humidified 5% CO_2_ atmosphere prior to testing. The serum-free medium was replaced with different material elutes. Cells cultured in α-MEM medium containing 10% FBS were the negative control. After 24, 48 and 72 h, MTT (1 mg/mL in CCM) was added after cell seeding and incubated for 4 h at 37 °C and 5% CO_2_. The MTT insoluble formazan was then dissolved by means of dimethyl sulfoxide DMSO that was applied for 2–4 h at 37 °C. The optical density (OD) was measured against blank (DMSO) at a wavelength of 570 nm (Abs570) and a reference filter of 690 nm by an automatic microplate reader (ELx800; Bio-Tek Instruments, Winooski, VT, USA)

#### 2.3.5. Cell Migration

To study the chemotactic effect of bleaching extracts, *h*DPSCs were seeded on 12-well plastic dishes and incubated in culture medium for 24 h until until a monolayer formed. A pipette tip was used to generate a cross-shaped scratch or “wound” and the cell debris was removed with PBS. Plated cells were incubated with various dilutions of the eluates up to 48 h to allow cell migration back into the wounded area. Images of the scratched areas were captured at 0, 24 and 48 h using a phase-contrast microscope (Nikon, Tokyo, Japan). Two images photomicrographs per well were takenat each indicated time. ImageJ software (NIH, Bethesda, MD, USA) was used to evaluate the wound closure area.

#### 2.3.6. Cell Morphology in Presence of Extracts

hDPSCs were plated on the glass coverslips at a low density and cultured in culture medium containing different material extracts to investigate morphological changes. The cells were fixed with 4% paraformaldehyde in phosphate-buffered saline (PBS) for 20 min at 2537 °C, permeabilized with 0.25% Triton X-100 (Sigma-Aldrich, St. Louis, MO, USA) for 3 min, and washed 3 times with PBS. For staining filamentous actin (F-actin), cells were incubated with CruzFluor594-conjugated phalloidin (Santa Cruz Biotechnology, Dallas, TX, USA). Nuclei were counterstained with 4,6-diamidino-2-phenylindole dihydrochloride (DAPI; Sigma-Aldrich). Fluorescent images were obtained using a confocal microscopy (Zeiss, Oberkochen, Germany).

#### 2.3.7. Analysis of the Expression of Mesenchymal Stem Cell Surface Markers on hDPSCs Exposed to Bleaching Products Using Flow Cytometry

The expression of mesenchymal stem cell surface molecules was analysed in cultures of *h*DPSCs in 2nd–4th passages using flow cytometry. Briefly, cells were seeded at a density of 3 × 10^4^ cells/cm^2^ in 48-well plates and treated with the different eluates for 72 h at 37 °C. Cells were detached using a 0.25% *w*/*v* trypsin-EDTA solution. This was then rinsed twice with PBS and incubated in the dark at 4 °C for 30 min with fluorescence-conjugated specific monoclonal antibodies for human CD73, CD90 and CD105 (MiltenyiBiotec, BergischGladbach, Germany), as recommended by the International Society of Cellular Therapy (ISCT), to confirm the mesenchymal phenotype of the cells (Dominici et al., 2006). The lack of expression of the hematopoietic markers CD14, CD20, CD34 and CD45 was also analysed. Non-specific fluorescence was measured using specific-isotype monoclonal antibodies. Cells were acquired using a BD FACS Canto flow cytometer (BD Biosciences) and analysed using Kaluza analysis software (Beckman Coulter, Inc., Brea, CA, USA).

#### 2.3.8. Detection of Apoptosis and Necrosis Using Flow Cytometry (Annexin-V/7-AAD Staining)

*h*DPSCs were cultured in different bleaching extracts for 72 h, which was followed by double staining with PE-conjugated Annexin-V and 7-AAD (BD Biosciences, Pharmingen). Percentages of live (Annexin-V^−^/7-AAD^−^), early apoptotic (Annexin-V^+^/7-AAD^−^) or late apoptotic and necrotic cells (Annexin-V^+^/7-AAD^+^ and Annexin-V^−^/7-AAD^+^) were determined using flow cytometry, before the percentages of each population were calculated.

### 2.4. Statistical Analysis

Two independent experiments were performed for all protocols in this study. Data were compiled and subjected to Levene’s test to verify the homogeneity of variance. Data data were compiled and subjected to one-way ANOVA and Tukey’s test (* *p* < 0.05, ** *p* < 0.01 and *** *p* < 0.001.)

## 3. Results

### 3.1. HP/CP Diffusion

**Figure 1:**
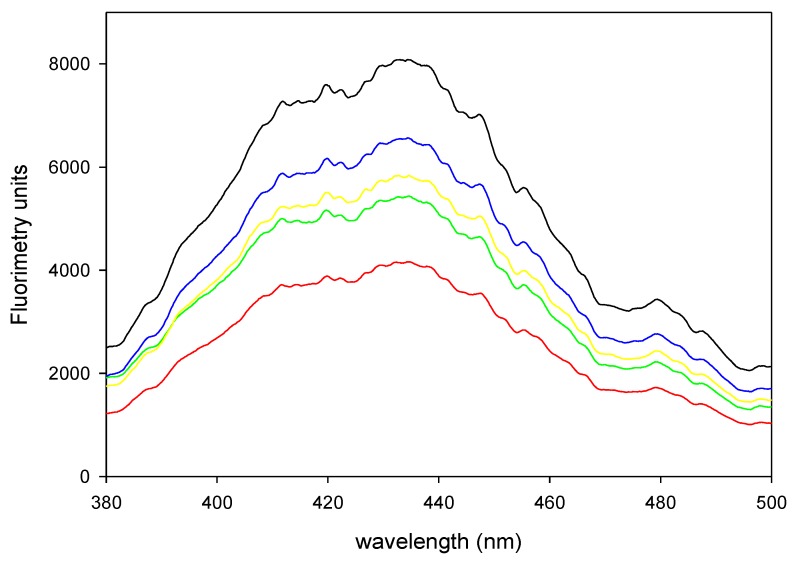
Fluorometric spectrum of samples of PO30 diluted 1:2 (black), PO3x15 diluted 1:4 (red), PB30 diluted 1:2 (green), PB3x15 diluted 1:4 (yellow) and PN90 diluted 1:2 (blue).

All bleached groups differed in HP diffusion. We created a 1:2 dilution of the productions of one application, while we created 1:4 dilutions of the products of 3 applications to obtain unsaturated measures of fluorometry. Our results demonstrated variation in the quantification of homovanillic dimer (HD) between bleaching products (Figure 1). Table 1 shows that HD concentrations with PN were lower than those with the other products despite the longer exposure time (90 min). Pola Office exhibited no differences with different numbers of applications. Perfect Bleach exhibited great diffusion with three applications.

### 3.2. Isolation and Characterisation of hDPSCs

**Figure 2:**
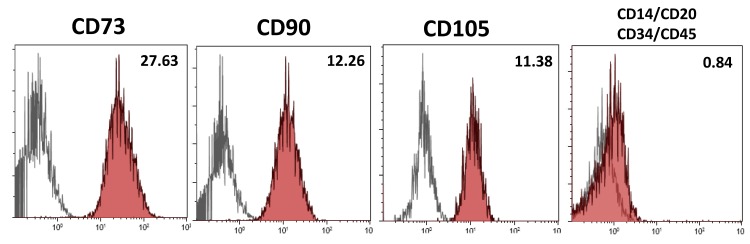
Immunophenotypic characterization of *h*DPSCs by flow cytometry for the expression of mesenchymal (CD73, CD90 and CD105) and hematopoietic (CD14, CD20, CD34 and CD45) stem cell (SC) markers (grey histogram: unstained control, red histogram: marker of interest). Results are means of triplicates of three independent experiments.

Positive cell surface marker expression of the mesenchymal markers CD73, CD90 and CD105 and negative expression of the hematopoietic markers CD14, CD20, CD34 and CD45 were evaluated on *h*DPSCs using flow cytometry. More than 95% of viable *h*DPSCs were positive for the mesenchymal markers CD73, CD90 and CD105 and negative for the hematopoietic markers CD14, CD20, CD34 and CD45 (Figure 2).

### 3.3. MTT Assays

**Figure 3:**
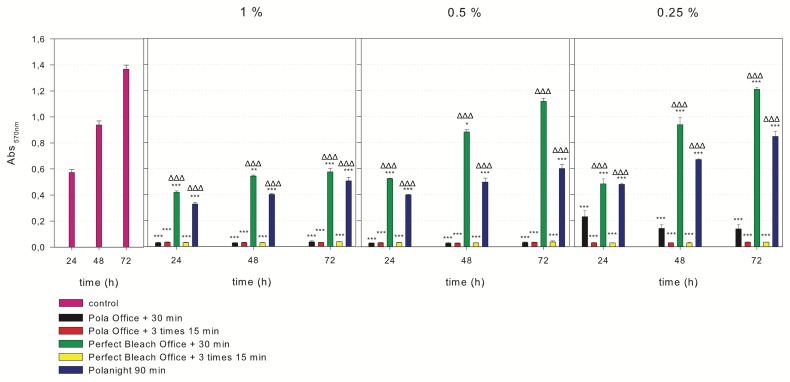
Cell viability was determined using the 3-(4,5-dimethyl-thiazol)-2,5-diphenyl-tetrazolium bromide (MTT) assay. The incubation of hDPSCs with 1% of each bleaching product revealed a significant reduction in cell viability rates compared with those in the control group at the indicated times of culture (*** *p* < 0.001). In contrast, a slight but significant recovery of cell viability was observed with 1%, 0.5% and 0.25% PB30 or PN compared to the others (^ΔΔΔ^ *p* < 0.001).

MTT assays were performed to evaluate the effects of the eluates of different bleaching agents on *h*DPSC viability (Figure 3). The incubation of *h*DPSCs with 1% of each bleaching product yielded a significant reduction in cell viability rates compared with the control group at the indicated times (*** *p* < 0.001). In contrast, the 1%, 0.5% and 0.25% PB30 or PN allowed a slight but significant recovery of cell viability compared to PO30, PO3x15 or PB3x15 (^ΔΔΔ^ *p* < 0.001). There was not difference in cell viability between 1 and 3 applications of the bleaching agents, except PO as there were significant differences in cell viability between 0.25% PO30 and PO3x15 (** *p* < 0.01). Our results revealed differences between bleaching agents with one application and PB30 produced better viability rates than PO30 and PN (*** *p* < 0.001).

### 3.4. Cell Migration

**Figure 4:**
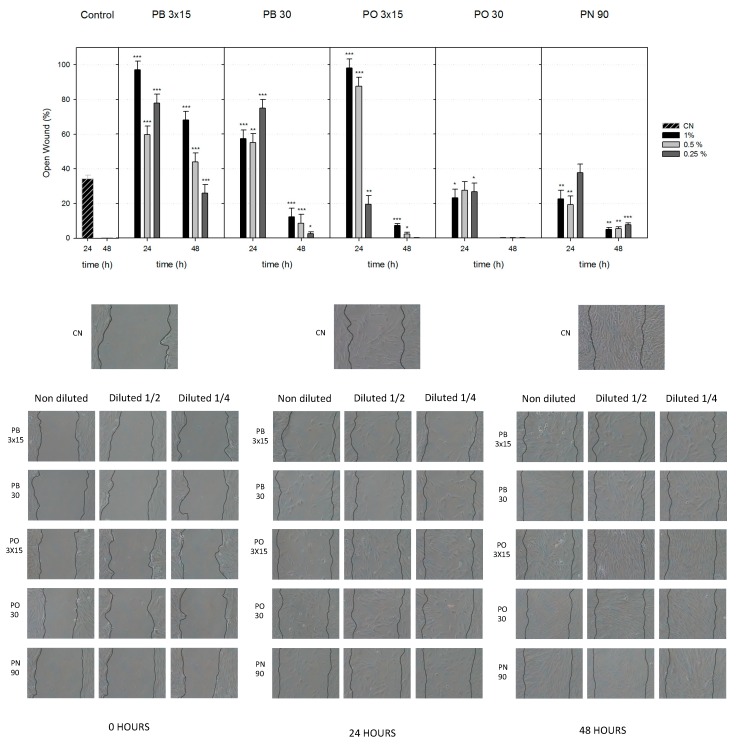
Wound-healing assay. Observation of wound closure change. Confluent hDPSCs were wounded and stimulated with bleaching extracts for up to 48 h. Cell migration is represented as the percentage of the open wound area under each condition compared with the control (* *p* < 0.05; ** *p* < 0.01; *** *p* < 0.001), which was analysed using one-way ANOVA.

Compared to treatment with the complete medium (control), treatment with the different dilutions of PB3x15, PB30 and PO3x15 (except 0.25%) for 24 h induced a significantly lower cell migration rate (** *p* < 0.01 and *** *p* < 0.001; Figure 4). Cell migration with PN and PO30 was still impaired but slightly higher at 24 h. Extracts of PO30 significantly promoted wound closure after 48 h of treatment and achieved comparable levels to control extracts. The PN group exhibited a slightly faster cell migration than the PB30 group, but both groups exhibited larger wound openings than the control group. Taken together, our results demonstrated that PO30 exerted a stronger effect on the migration ability of *h*DPSCs, while PN and PB30 produced values that were similar to those of the controls.

### 3.5. Cell Morphology

**Figure 5:**
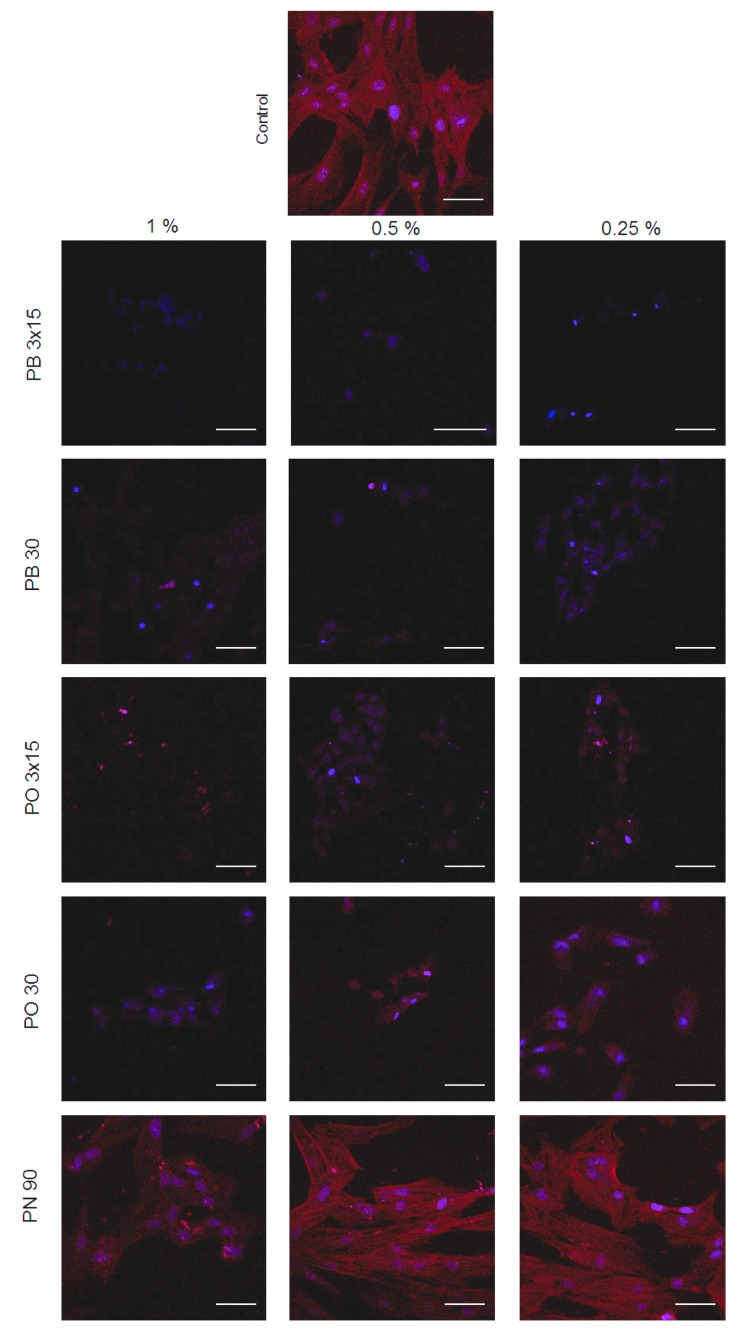
Representative immunofluorescence micrographs revealing the cytoskeletal organisation of *h*DPSCs exposed to control and bleaching products. Cultured *h*DPSCs were treated with undiluted extracts of bleaching products for 24 h. For staining filamentous actin (F-actin), cells were incubated with CruzFluor594-conjugated phalloidin (Santa Cruz Biotechnology, Dallas, TX, USA). Nuclei were counterstained with 4,6-diamidino-2-phenylindole dihydrochloride (DAPI) (blue). Scale bar = 150 μm.

The immunofluorescence assay was performed to detect changes in cellular morphology and cytoskeletal organisation of hDPSCs by phalloidin (red fluorescence) and DAPI (blue fluorescence). hDPSCs without bleaching extracts (control) showed a gradual increase in cell proliferation with fibroblastic morphology, a high expression of F-actin and confluency after 72 h of culture (Figure 5). Notably, treatment with extracts of PB3x15, PB30, PO3x15 and PO30 resulted in a small proportion of cells in both groups that lacked F-actin cytoskeleton staining and exhibited condensed or fragmented nuclei, which is typical of apoptotic cells. PN, especially at 0.25% and 0.5% concentrations, exhibited a similarly organised and stretched stress assembly of fibers compared with controls, which provides evidence for the optimal status of *h*DPSCs.

### 3.6. Analysis of the Expression of Mesenchymal Stem Cell Surface Markers on hDPSCs Exposed to Bleaching Extracts Using Flow Cytometry

**Figure 6:**
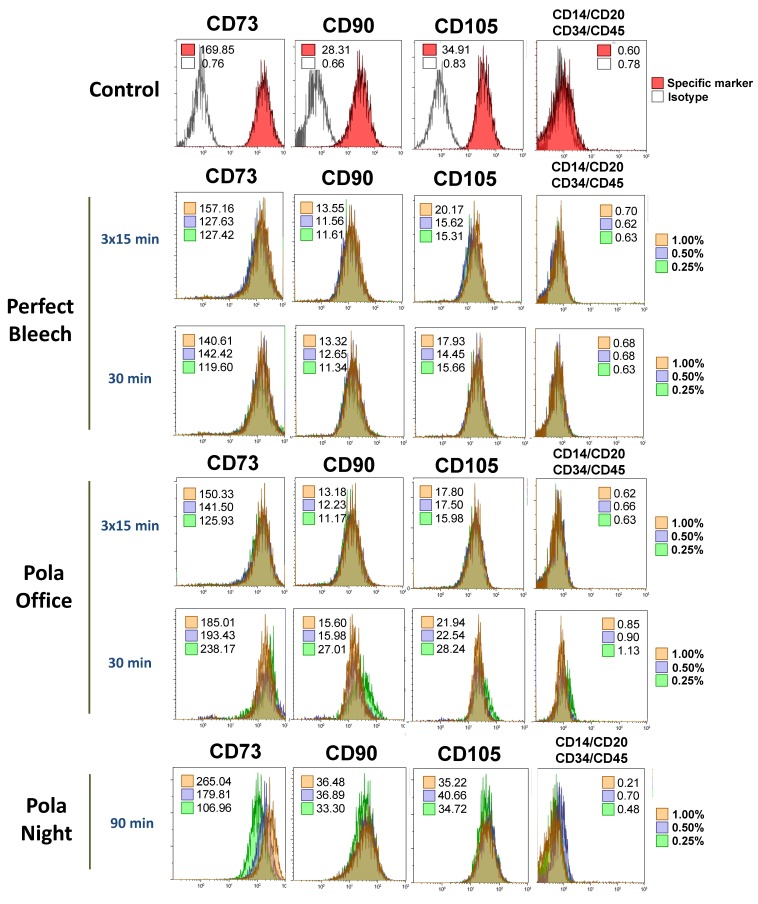
Mesenchymal phenotype analysis of *h*DPSCs after culture with bleaching products using flow cytometry. Cells were cultured for 72 h, detached and labelled with fluorescence-conjugated specific antibodies for the mesenchymal surface markers CD73, CD90 and CD105 and the hematopoietic markers CD14, CD20, CD34 and CD45. Inset numbers represent the mean fluorescence intensity values of viable cells. Histograms show representative flow cytometry results from three independent experiments.

Flow cytometry studies were performed to determine possible phenotypic changes in viable *h*DPSCs after culture with the eluates of the different bleaching agents. MSC surface molecules CD73, CD90 and CD105 were expressed at levels greater than 95%, while the expression level of the hematopoietic markers CD14, CD20, CD34 and CD45 was lower than 5% (Figure 6). Notably, the incubation of *h*DPSCs with different dilutions of the extracts (1%, 0.5%, or 0.25%) did not significantly alter the percentage of positive expression of these mesenchymal markers compared to that in untreated *h*DPSCs (controls).

### 3.7. Apoptosis/Necrosis of hDPSCs in the Presence of Bleaching Extracts

**Figure 7:**
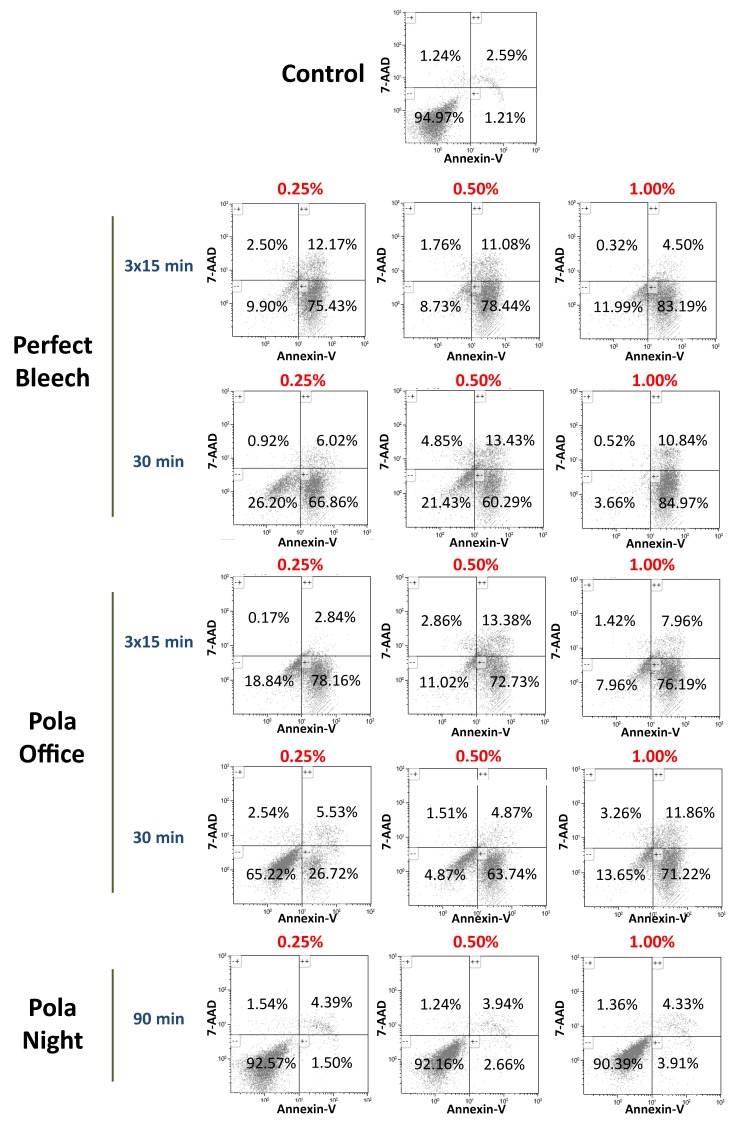
*h*DPSCs were cultured with bleaching extracts and plastic (control) for 72 h, before being labelled with Annexin-V and 7-AAD and analysed using flow cytometry. Numbers within the different quadrants represent the percentages of live (Annexin-V^−^/7-AAD^−^), early apoptotic (Annexin-V^+^/7-AAD^−^) or late apoptotic and necrotic (Annexin-V^+^/7-AAD^+^ and Annexin-V^−^/7-AAD^+^) cells. Dot-plots display representative flow cytometry results from three independent experiments. comma in the figure should be decimal point.

Figure 7 shows the representative 2D dot plots of the distribution of viable (Annexin-V^−^/7-AAD^−^), early apoptotic (Annexin-V^+^/7-AAD^−^) or late apoptotic/necrotic (Annexin-V^+^/7-AAD^+^ and Annexin-V^−^/7-AAD^+^) cells among the untreated *h*DPSCs (controls) or cells exposed to different dilutions of the bleaching extracts. The PN extract was associated with more than 90% of the cells remaining viable after 72 h. In contrast, the more concentrated eluates (1% and 0.5%) of the other bleaching agents decreased cell viability. This cytotoxic effect was minor in the most diluted extracts (0.25%).

## 4. Discussion

To our knowledge, this is the first study to compare commercial bleaching products and their biological effects on stem cells from dental pulp. Little information on the diffusion capacity of commercial bleaching products and their effects on dental pulp tissue is available. Therefore, the present study quantified the diffusion of commercial bleaching products and their influence on the biological responses of hDPSCs.

The trans-enamel and trans-dentinal diffusion of bleaching products was based on a previously reported method [24], which is more sensitive than spectrophotometry. Our results demonstrated a higher diffusion of bleaching products after 3 applications. These results corroborate previous studies, which revealed that three applications of gels with greater HP concentrations produced greater diffusion of HP to the pulp chamber and increased damage to the dental pulp [11,12]. Therefore, the exposure time was less important than the number of applications. Our results demonstrated that PN (90 min) exhibited lower rates of diffusion than the other bleaching products. This is the less concentrated product as 16% CP is equivalent to 5–6% HP, but its diffusion was similar to that obtained with PB30.

Pulpal inflammation associated with local tissue necrosis was demonstrated in vivo by the application of high-concentration HP bleaching gels (35–38%) to human teeth [7,29]. hDPSCs are used as the cell line model to simulate clinical conditions because these cells play a major role in dental pulp inflammation and tooth hypersensitivity [25,30].

We analysed the biological cell responses of hDPSCs with respect to cell viability, cell migration, phenotype, apoptosis and morphology in the presence of three commercial bleaching products. The present investigation demonstrated that the PB3x15 and PO3x15 bleaching gel applications were cytotoxic to *h*DPSCs because cell viability was reduced. These results are clinically interesting because the number of applications may be involved with the occurrence of tooth sensitivity. In fact, Benetti et al. [31] demonstrated that major damage to rat dental pulp occurred after three applications of an HP gel, while de Oliveira et al. [6] also observed a significant reduction in cell viability following three applications of 10% HP gel for 15 min.

Previous studies demonstrated the healing of damaged pulp via the formation of a hard-tissue barrier or reparative dentine. The migration of pulp stem cells to substitute for irreversibly damaged odontoblasts after differentiation is an important step in this process [25]. In general, three applications of the bleaching gel produced lower cell migration than PN or PO30. However, we observed better cell migration in Pola Office groups than in Perfect bleach groups. Notably, Vaz et al. [32] reported that pulp inflammation was related to increased macrophage migration. These authors observed higher macrophage migration with in-office bleaching compared to at-home bleaching.

Optimising the state of dental pulp stem cells contributes to cell migration and dental pulp repair [21]. Immunofluorescence staining showed that undiluted extracts of cells treated with PN produced no morphological alterations and no changes in cytoskeletal organisation patterns compared to cells treated with the other bleaching products. Again, we observed better cell morphology in PO30 than PB30.

Analysis of the expression of mesenchymal stem cell markers is important in regenerative dentistry [33], which was performed in this study. The ISCT states that multipotent mesenchymal stromal cells must express CD105, CD73 and CD90 and should be devoid of the expression of hematopoietic markers, such as CD45, CD34, CD14 and CD11b [26]. We characterised the surface expression pattern of these markers using flow cytometry to evaluate possible changes in the expression. With commercial bleaching products, the MSC surface molecules CD73, CD90 and CD105 were expressed at levels greater than 95% following commercial bleaching, while the expression level of the hematopoietic markers CD34 and CD45 was less than 5%. Therefore, the bleaching products used in this study maintained the mesenchymal phenotype of hDPSCs.

Apoptosis plays an important role in the formation of reparative dentin by providing room for new dentin and preventing inflammation. Apoptosis also leads to the formation of mature dental pulp when exposed to extraneous stimuli [34]. Our results revealed that PN did not induce apoptosis, which is consistent with the results from Benneti et al. [31] as they demonstrated that the effects on pulp tissue varied with the HP concentration. These authors observed that higher HP concentrations produced pulp necrosis and had a prolonged effect on the apoptotic process, while lower HP concentrations induced moderate inflammation, cell proliferation and apoptosis. Nevertheless, in the present study, also in the apoptosis experiment, bleaching products with 35% HP concentration were more cytotoxic than 37% HP and caused membrane permeability-related apoptosis and necrosis. Thus, this effect is more dependent on the commercial product than on the composition.

## 5. Conclusions

In general, a low concentration of bleaching products, such as PN applied for 90 min, was less cytotoxic than other commercial bleaching products with 35–37.5% HP concentration. This effect occurred independently of the application protocol. For high concentration products, bleaching products with 35% HP concentration were more cytotoxic than 37% HP, which suggests that unknown agents in bleaching products could play an important role in determining their level of toxicity.

## Figures and Tables

**Table 1:** Bleaching products diffusion.

Bleaching Products	Applications	Diffusion (mM)
Pola Office	1 × 30	38.4 ± 8.2
Pola Office	3 × 15	39.3 ± 1.2
Perfect Bleach	1 × 30	28.2 ± 6.5
Perfect Bleach	3 × 15	48.6 ± 1.7
Polanight	1 × 90	27.7 ± 0.4
